# Highlights of the 2020 23rd Society for Cardiovascular Magnetic Resonance Scientific Sessions

**DOI:** 10.1186/s12968-020-00672-6

**Published:** 2020-10-29

**Authors:** Lars Grosse-Wortmann, Christopher J. Francois, Lilia M. Sierra-Galan, Michael Markl, Javier Sanz, James Carr, Chiara Bucciarelli-Ducci, Andrew J. Powell

**Affiliations:** 1 Division of Cardiology, Department of Pediatrics, Oregon Health and Science University, Portland, OR USA; 2 Department of Paediatrics, The Hospital for Sick Children, University of Toronto, Toronto, ON Canada; 3 Department of Radiology, Mayo Clinic, Rochester, MN USA; 4 American British Cowdray Medical Center, Mexico City, Mexico; 5 Department of Radiology, Feinberg School of Medicine, Northwestern University, Chicago, IL USA; 6 Department of Biomedical Engineering, McCormick School of Engineering, Northwestern University, Evanston, IL USA; 7 Icahn School of Medicine At Mount Sinai, New York, NY USA; 8 Department of Radiology, Feinberg School of Medicine, Northwestern University, Chicago, IL USA; 9 Clinical Research and Imaging Centre, University of Bristol, Bristol, UK; 10 NIHR Biomedical Research Centre, University Hospitals Bristol NHS Foundation Trust, Bristol, UK; 11 Department of Cardiology, Boston Children’s Hospital Department of Pediatrics, Harvard Medical School, Boston, MA USA; 12 Doernbecher Children’s Hospital, CDRC, 707 SW Gaines Street, Portland, OR 97239 USA

## Introduction

**Fig. 1:**
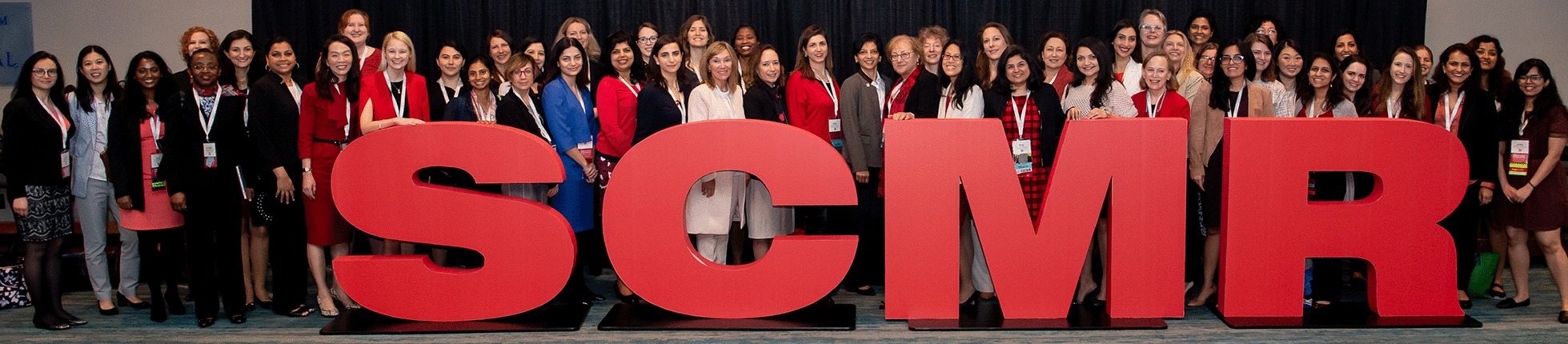
The lobby was a favorite locale for networking and socializing with friends and colleagues over refreshments

**Fig. 2:**
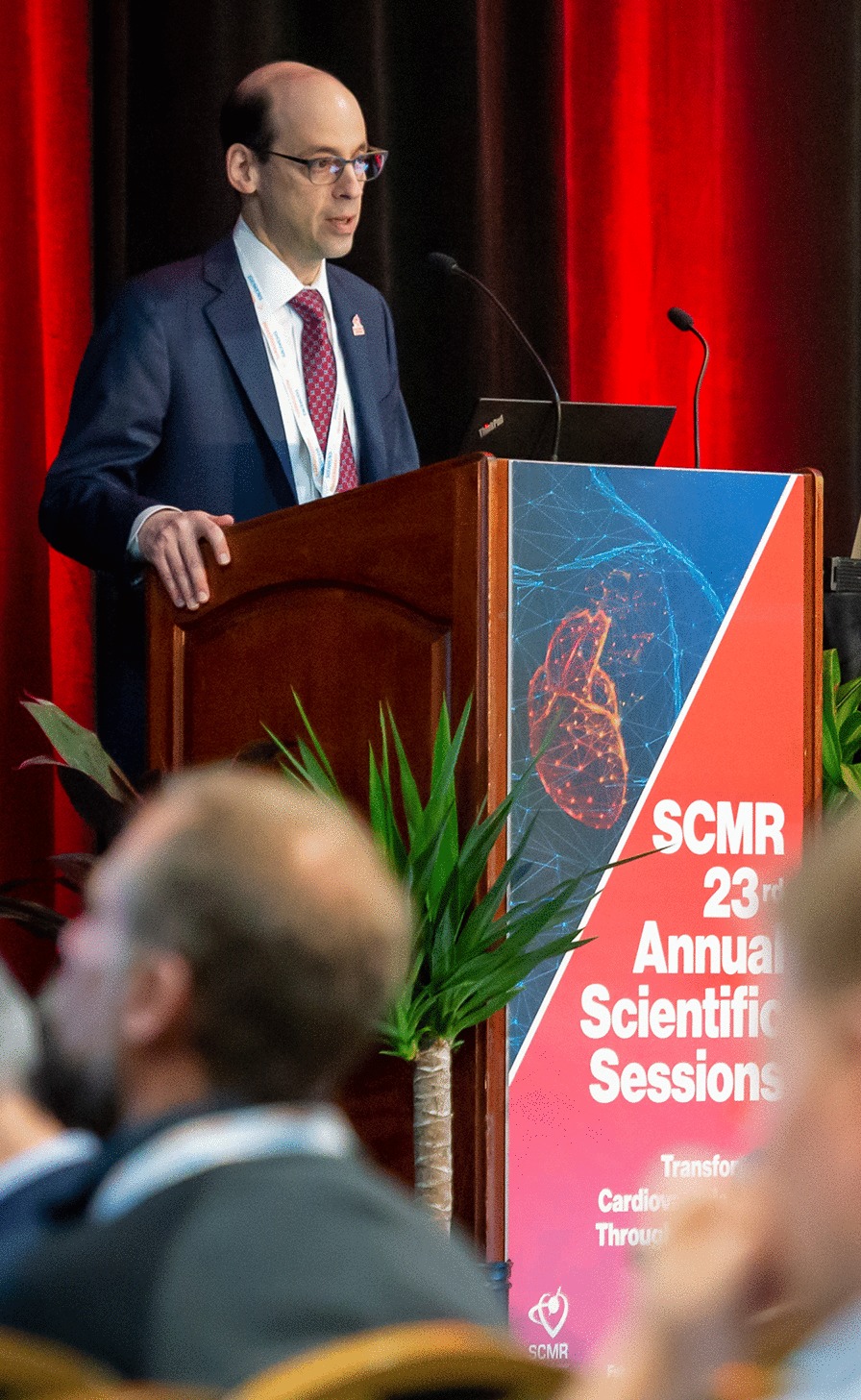
President Dr. Andrew Powell opens the 23rd Annual SCMR Scientific Sessions in Orlando, Florida, USA

Cardiovascular magnetic resonance (CMR) is an integral part of the evaluation and management of patients with cardiovascular disease. As a result, the interest in this modality among scientific and clinical communities continues to grow, as evidenced by a record number of attendees at the 23rd Society for Cardiovascular Magnetic Resonance (SCMR) Annual Scientific Sessions. More than 1,440 delegates from around the world experienced the meeting in Orlando, Florida, USA from February 12–15, 2020 (Fig. 1). Two thirds of attendees were from North America and a fifth from Europe. Cardiologists constituted the most prevalent professional designation which is a testament to the important role of CMR in routine clinical cardiology practice and cardiology research. The theme of the meeting was entitled, “Transforming Cardiovascular Care Through Discoveries in Imaging” (Fig. 2). A total of 112 sessions spanned the spectrum from technical developments to basic discoveries, clinical science and advocacy. The meeting was preceded by a joint workshop together with the International Society for Magnetic Resonance in Medicine (ISMRM) and several preconference courses, including a general overview of CMR techniques and applications for physicians, a course around pediatric and congenital heart disease, and a review of the fundamentals of cardiology. The theme of the SCMR/ISMRM workshop was “CMR 4.0: Autonomous and Efficient Cardiovascular Magnetic Resonance Imaging”. This 1.5 day event focused on the topics of ‘efficient and effective CMR’, ‘autonomous scanning and quantification’, ‘hardware innovations’, as well as ‘artificial intelligence (AI)’ and ‘big data’. A complete course on interventional CMR was also offered. Fifty-seven abstract and didactic sessions during the main meeting highlighted recent CMR technical and clinical developments as well as scientific discoveries. These ranged from improving quantitative precision for diagnosis of cardiovascular disease in an individual patient to identifying methods of using CMR to improve outcomes. Within this context, sessions showcased work from around the world using CMR in congenital heart disease, ischemic and non-ischemic heart disease, valvular disease, electrophysiology, systemic inflammation, cardio-oncology, heart failure, and vascular pathology. This report highlights a few of the contributions in the areas of ‘Innovative Clinical Applications of CMR’, ‘Clinical and Translational Science’, and ‘Basic Science & Technical Developments’. Table 1 lists the scientistis, clinicians, and abstracts that were specifically recognized during the meeting.

**Table 1:** Award winners during the 23rd SCMR Scientific Sessions

Award	Awardee	Abstract title
Gold medal	Jeanette Schulz-Menger (Fig. 3a)	
Gold medal	Peter Kellman (Fig. 3b)	
Early Career Award—Clinical Science	Sarah Ghonim	Independent and relative value of late gadolinium enhancement in predicting compromising ventricular arrhythmia or mortality in adults with repaired tetralogy of Fallot
Early Career Award—Basic Science and Technical Developments	Tushar Kotecha	Assessment of ischaemic burden in multi-vessel coronary artery disease using CMR pixelwise quantitative perfusion mapping
Best moderated e-poster	Austin Robinson	Diagnostic accuracy of high resolution stress myocardial perfusion imaging with whole heart coverage at 3 T
Best technologist abstract	Bao Ru Leong (Fig. 4)	Cardiac mass or aneurysmal saphenous vein graft?
Best case of the week	Arun Dahiya	Early diagnosis of a systemic disease by CMR prevents complications
Seed Grant	Nivedita Naresh (Fig. 5a)	CMR for cardiotoxicity in kids
Seed Grant	Allen Bradley (Fig. 5b)	Evaluating multi-institution variability in 4D flow hemodynamic characterization of Type B aortic dissection with 3D printed models

**Fig. 3:**
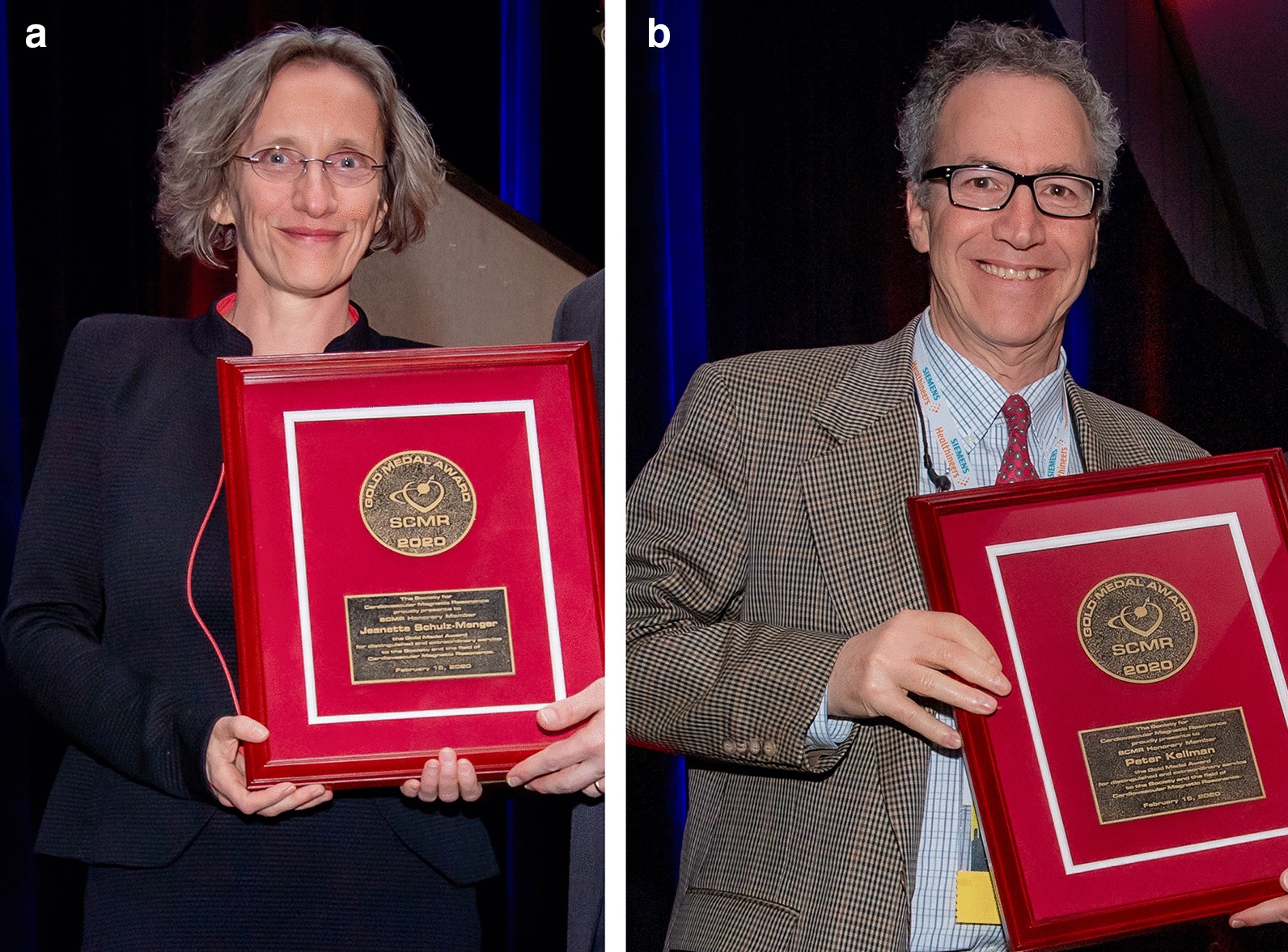
Drs. Jeanette Schulz-Menger (**a**) and Peter Kellman (**b**) were awarded the 2020 SCMR Gold Medal Awards

**Fig. 4:**
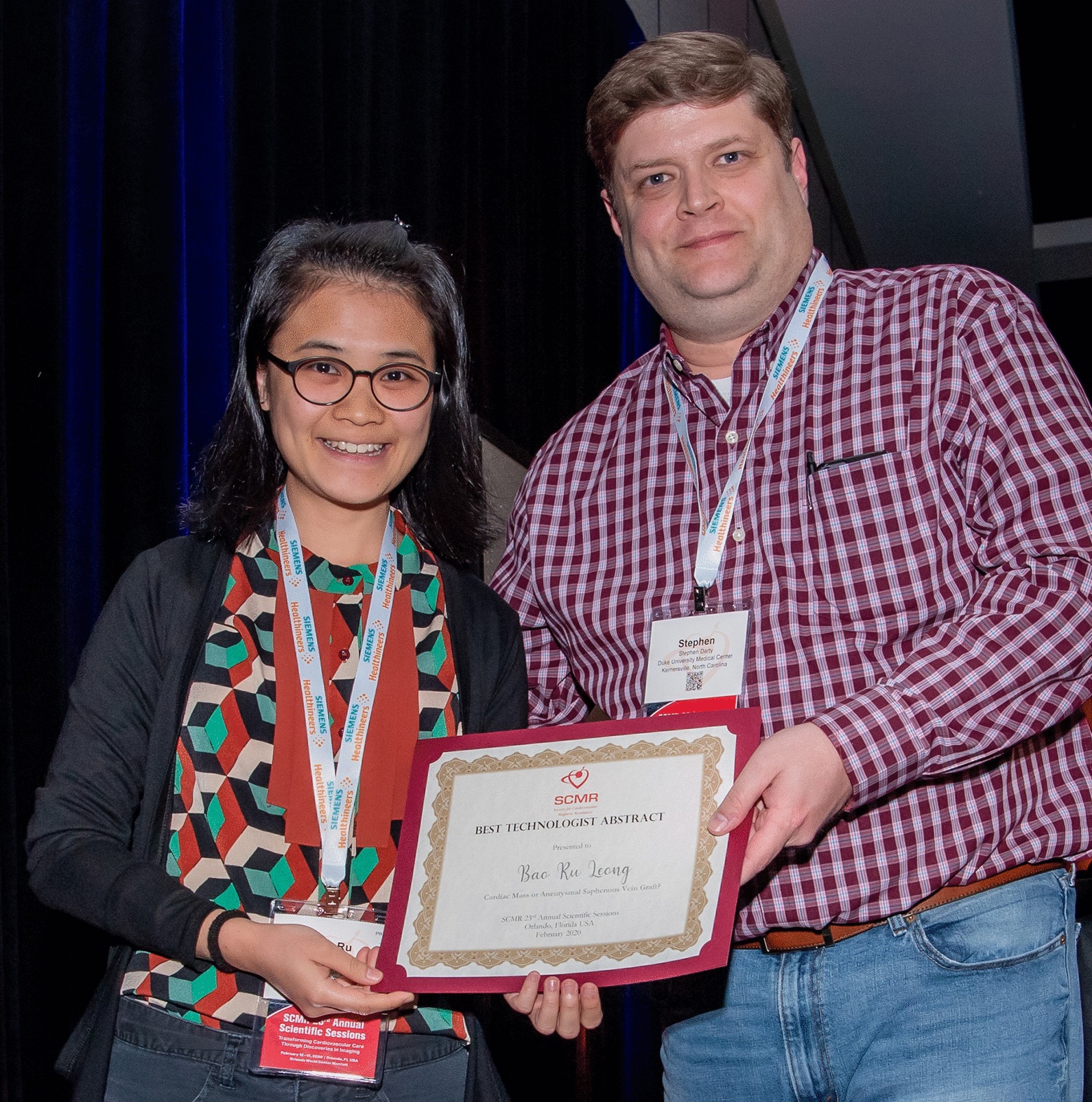
Bao Ru Leong (left) submitted the best technologist abstract

**Fig. 5:**
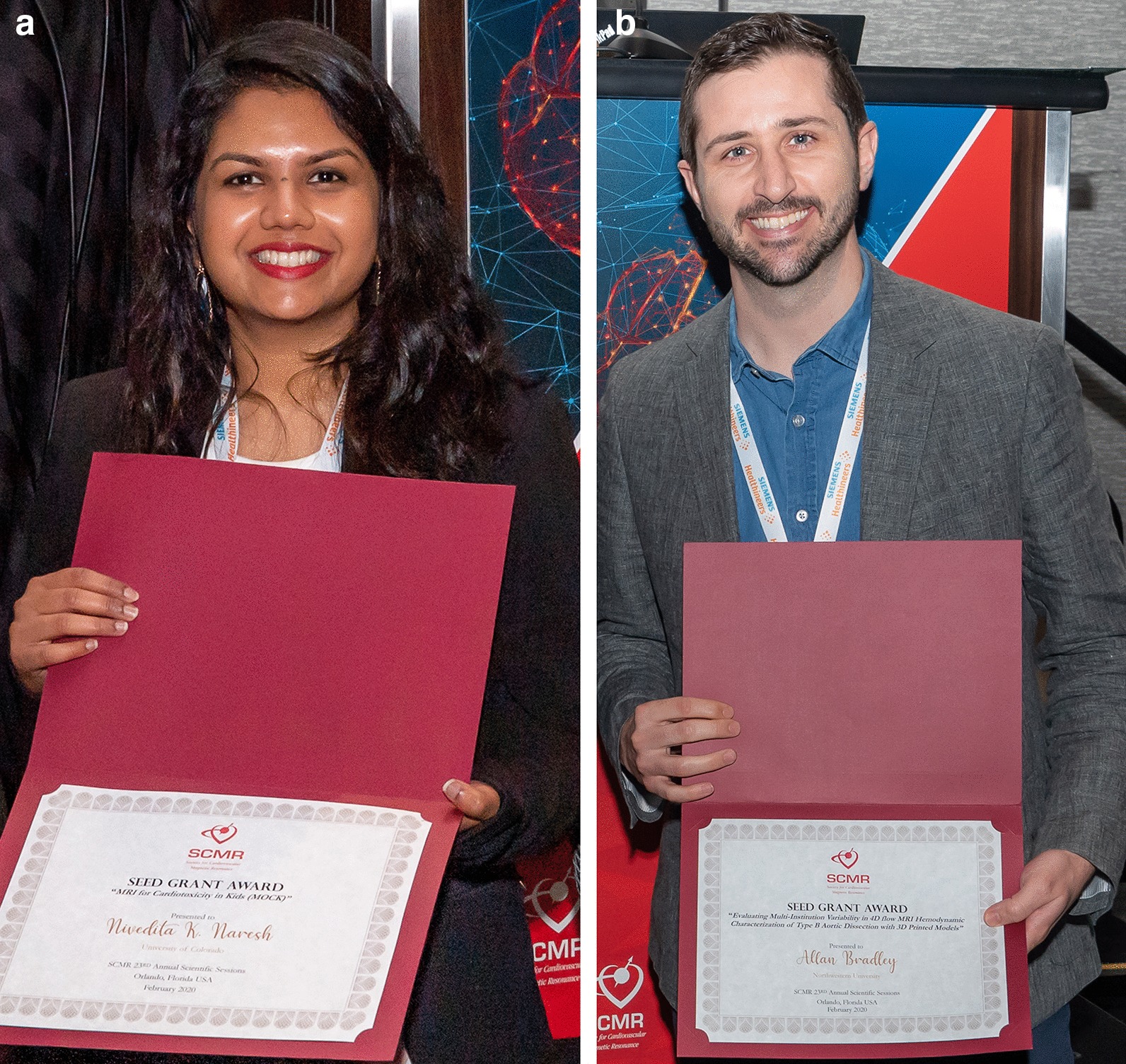
Drs. Nivedita Naresh (**a**) and Allan Bradley (**b**), winners of the 2020 SCMR Seed Grant Awards

## Innovative clinical applications of CMR

The 2020 SCMR Scientific Sessions covered a wide arena of clinical aspects of CMR. Themes were AI in imaging, stress CMR, discoveries from large cohorts and registries, congenital heart disease, and cost-effectiveness analyses.

AI has become a reality in all aspects of medicine. Diagnostic imaging is at the forefront of its application and was on display for CMR throughout the meeting. Visionary plenary lectures by Drs. Anthony Chang and Michael McConnell set the tone for the meeting and explored potential and real impacts of AI on scanning, post-processing, reporting, diagnosis, and risk stratification. Hann et al. [1] introduced a novel quality control-driven framework as a solution not only to automatically contour T1 maps but also to quality-check the contours. In a study with > 1900 patients from 14 centers, Davies et al. [2] showed that machine learning can exceed human performance not only for speed, but also for quality in measuring left ventricular (LV) volumes and ejection fraction (LVEF). They found that the automatic analysis resulted in improved reproducibility of ventricular volumes compared to human observers. Further, machine learning derived measurements were superior in predicting patient outcomes. Mohan et al. [3] found that training machine learning algorithms in single ventricle congenital heart disease accelerates and improves the accuracy of ventricular contouring.

During his plenary talk, former SCMR chief executive officer and 2019 SCMR Gold Medal awardee Dr. Orlando Simonetti illustrated the feasibility of acquiring myocardial perfusion images during exercise using CMR compatible treadmills and supine ergometers. LaFountain et al. [4] reported that subambient oxygen concentrations during an exercise stress perfusion CMR increased the likelihood of achieving submaximal heart rates.

Large multicenter cohorts and registries are increasingly used to demonstrate the role of CMR in risk prediction for cardiovascular disease: The SCMR Registry [5], launched in 2013, continues to bear fruit. Antiochos et al. [6] presented follow-up data from the Stress CMR Perfusion Imaging in the United States (SPINS) registry in 2,349 individuals with suspected myocardial ischemia. An abnormal stress perfusion CMR correctly reclassified the risk of cardiovascular death or non-fatal myocardial infarction (MI), particularly in the intermediate-risk category. Two studies tested the prognostic value of stress perfusion CMR in ischemic heart disease: Pezel et al. [7] reported increased risk of death or MI in 1,049 patients with heart failure and reduced LVEF who showed signs of myocardial ischemia on vasodilatory stress CMR. Further, Kinnel et al. [8] demonstrated the ability of the same modality to predict adverse cardiovascular events in 866 patients following surgical coronary revascularization. Employing an operator-independent, AI-based approach for absolute perfusion quantification, Knott et al. [9] demonstrated in 1,049 patients that reductions in or myocardial perfusion reserve predicted future death or major cardiovascular events. The Cardiovascular Imaging Registry of Calgary (CIROC) [10] confirmed that caffeine, alcohol, and soda consumption are related to major adverse cardiovascular outcomes, even after adjusting for disease phenotypic features on CMR imaging. The same effort [11] identified CMR-derived left atrial volume as a risk factor for de novo atrial fibrillation in patients with cardiovascular disease. Cornhill et al. [12] followed 737 patients with non-ischemic dilated cardiomyopathy who underwent CMR. A composite score containing six clinical variables (age, diabetes, functional class, use of anticoagulants, digoxin, or diuretics) and three CMR-derived parameters [LVEF, hypertrophy, and midwall late gadolinium enhancement (LGE)] performed well in risk stratifying for death or heart failure hospitalization.

For decades, CMR has been an integral part of clinical congenital heart disease care. Beroukhim et al. [13] presented a refinement of their landmark paper on cardiac masses in children, and, in an invited presentation, Hughes et al. [14] demonstrated the use of CMR in the preoperative evaluation of patients with Epstein’s anomaly.

Another focus during the meeting resided in cost-efficiency analyses. A study across 12 countries by Moschetti et al. [15] found that the use of CMR plus conventional angiography in patients with coronary artery disease reduced cost by an average of 39% versus an angiography plus fractional flow reserve strategy. Pandya et al. [16] introduced an easy to use value calculator that allows one to quickly compute the cost-effectiveness of CMR in health care settings around the world based on population characteristics and the amount billed. Kelle [17, 18] described an approach to increase access to CMR via a mobile scanner platform and remote readings.

## Clinical and translational science

Highlights in this area fall into five main groups: (1) the role of CMR following MI, (2) CMR augmentation of electrophysiology procedures, (3) serial CMR for the evaluation of therapeutic agents, (4) CMR in congenital heart disease, and (5) technical developments on the verge of clinical translation.

Kochav et al. [19] investigated the potential role of ischemia in the development of functional mitral regurgitation in a registry of 8,631 patients. Perfusion abnormalities and/or MI of the basal to mid anterior, lateral, and inferior walls were linked to functional mitral regurgitation, which in turn predicted mortality. Ischemia of sub-papillary myocardium was independently associated with regurgitation even after adjusting for presence of LGE or papillary muscle ischemia, suggesting a potential role in the pathogenesis of valve dysfunction. Schuster et al. [20] studied the prognostic role of right atrial function after acute MI. The authors found that the risks of reinfarction, death, or heart failure were associated with impaired right atrial reservoir, conduit, and booster functions, independent of right ventricular (RV) ejection fraction and atrial fibrillation.

The necessity of gadolinium-based contrast use was addressed by many contributions throughout the meeting, such as the spirited debate on this topic between Drs. Raymond Kim and Matthias Friedrich. Moreover, Nakamori et al. [21] demonstrated that the comparison of myocardial native T1 values during stress vs. rest can detect the physiological changes in the myocardium induced by supine exercise. Imaging with the calcium analogue manganese holds the promise of visualizing myocardial calcium-handling and therefore directly depicting viability in ischemic and non-ischemic cardiomyopathy: Spath et al. [22, 23] showed the utility of manganese-enhanced CMR for viability assessment in acute MI.

Bilchick et al. [24] presented early data of an ongoing randomized controlled trial comparing implantation of cardiac resynchronization devices using a standard versus an image-guided strategy. The latter, which included evaluation of temporal mechanical activation using 3D-displacement encoding with stimulated echoes, scar visualization with LGE, and coronary venous anatomy by computed tomography, allowed the avoidance of scarred myocardium for lead placement in all patients (versus 60% in the standard arm) and resulted in greater QRS shortening. Gulhane et al. [25] illustrated how LGE images can improve the ability to arrhythmogenic foci within the myocardium of patients with nonischemic cardiomyopathy.

In the arena of non-ischemic cardiomyopathies several studies highlighted the role of CMR mapping in monitoring therapies: Chacko et al. [26] prospectively examined the effects of patisaran on cardiac amyloid burden, ventricular function, and extracellular volume (ECV) in patients with transthyretin amyloidosis, in comparison with controls. The authors found that amyloid burden decreased, myocardial ECV decreased, and exercise tolerance improved in the treatment group, all in contrast to the observations in the control group.

In the field of congenital heart disease, Ghonim et al. [27] presented a large series of 550 patients with tetralogy of Fallot who underwent CMR and were followed for major arrhythmic events (ventricular tachycardia, ventricular fibrillation, or sudden cardiac death). A score containing clinical and imaging information reflecting scar burden and RV systolic function was able to classify patients into low-intermediate and high-risk groups with event rates ≤ 1% and 4.4%/year, respectively, Fig. 3). The authors submitted that the risk in the latter group may be high enough to consider defibrillator implantation for primary prevention based on CMR. Ven et al. [28] described an association between atrial function during dobutamine stress CMR and peak oxygen uptake in Fontan patients. Juffermans et al. [29] presented findings from a multicenter study which found good internal consistency of flow quantification with 4D flow CMR.

Finally, two studies presented early clinical results using CMR technologies that may soon be ready for more widespread applications: In patients with cryptogenic stroke, Soulat et al. [30] demonstrated beneficial effects of angiotensin-converting enzyme inhibitors or angiotensin receptor blockers on aortic stiffness and flow reversal in the descending aorta (a potential mechanism for stroke), using 4D flow CMR. Varghese et al. [31] tested a novel T2-mapping-based method for noninvasive quantitative cardiac blood oximetry that accounts for hematocrit and peripheral oxygen saturation and showed good agreement with invasive oximetry values, obtained by right heart catheterization.

## Basic science and technical developments

Numerous innovations were presented with a focus on (1) autonomous and multi-dimensional CMR, (2) interventional and low field CMR, (3) new contrast mechanisms, and (4) validation of techniques.

In the domain of autonomous multi-dimensional CMR, Munoz et al. [32] demonstrated the feasibility of efficient high-resolution 3D water/fat LGE atrial wall imaging in less than four minutes. The short acquisition time was achieved by highly accelerated 3D radial sampling combined with 2D image navigator motion estimation, resulting in 100% respiratory scan efficiency, and under-sampled multi-contrast reconstruction. In a pilot study, this approach reduced scan time by more than 50% while providing complementary water/fat images of the left atrium and reducing motion artifact compared to conventional 3D LGE imaging. In the same category, Hu et al. [33] presented a novel comprehensive multitasking-based assessment of the thoracic aorta. Within 6 min, they acquired an isotropic dataset without the use of electrocardiographic gating or diaphragm navigators that allowed the reconstruction of multi-contrast images (bright/dark/gray blood) in different cardiac and respiratory frames as well as in cine mode.

In the arena of interventional and low field CMR, a study by Greer et al. [34] demonstrated the potential of selective CMR guided contrast injection for the detection of arteriovenous malformations. In a direct comparison of imaging at 0.55 T and 1.5 T, Bandettini et al. [35] reported comparable balanced steady-state free precession cine image quality at the two field strengths, as long as sequence parameters at low field strength were optimized (reduced bandwidth and increased flip angle). Their results suggest that the advantages of low field CMR in the reduction of image artifacts and radiofrequency energy deposition can be harnessed while preserving image quality.

A new CMR contrast mechanism was presented by van den Boomen et al. [36] who reported on dynamic cardiac blood-oxygen-level dependent (BOLD) imaging with gradient-echo-spin-echo echo-planar imaging readout and simultaneous multi-slice radio frequency excitation. This approach acquired T2 and T2* changes during a breath-hold perturbation simultaneously at two short-axis slices. They found different BOLD responses at the two locations and speculated that these differences in vascular physiology could be exploited in ischemia evaluations. Phipps et al. [37] introduced residual denoising in order to accelerate in vivo diffusion tensor CMR: The authors trained a deep learning network to identify and then remove noise from the image. This allowed them to accelerate the image acquisition by the factor of two while maintaining sufficient signal-to-noise ratio and image quality to assess myocardial tissue parameters such as mean diffusivity, fiber angle, and helix angle.

Other important contributions included the validation of CMR techniques in experimental settings: Kotecha et al. [38] showed that CMR pixelwise quantitative perfusion mapping improved the detection of multi-vessel coronary artery disease in comparison with visual analysis (Fig. 4). A large animal study by Bradley et al. [39] underscored the diagnostic value of CMR quantitative perfusion mapping. The CMR derived mean blood flow ratio predicted invasive fractional flow reserve with high sensitivity and specificity for all 3 commonly used quantification methods. Using femoral tonometry as a gold standard, Nguyen et al. [40] tested the accuracy of different CMR methods for the assessment of vascular stiffness: Pulse wave velocity derived from 4D flow CMR performed best in differentiating patients from controls, with a sensitivity and specificity of 89% and 94%, respectively. Auger et al. [41] demonstrated excellent inter-user, inter-site, and inter-session reproducibility of DENSE for quantification of regional LV segmental circumferential strain.

## Conclusions

**Fig. 6:**
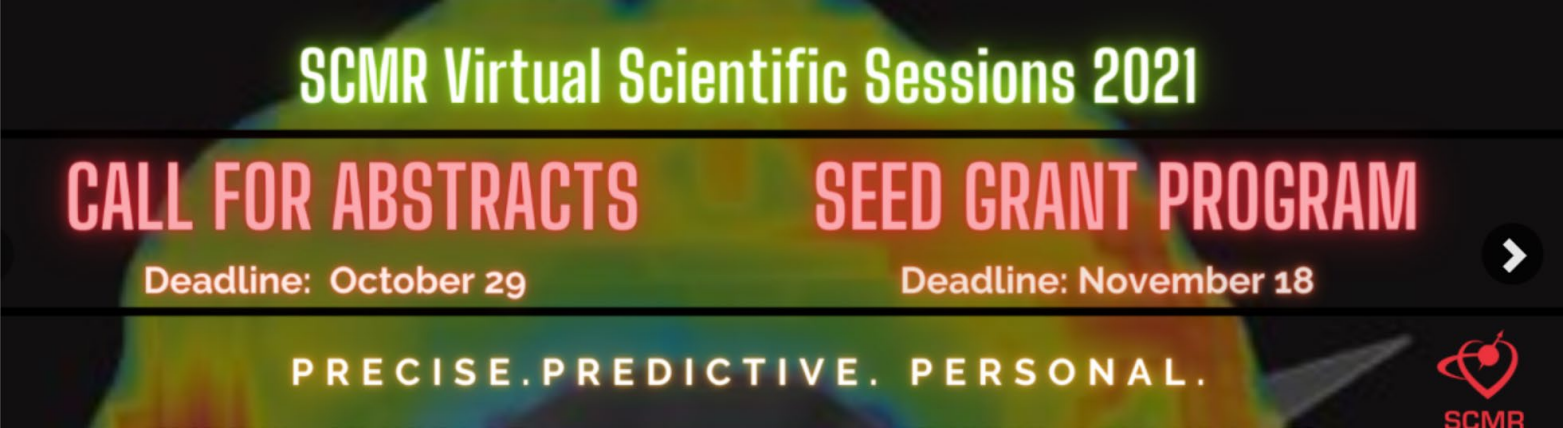
“Save the Date” for SCMR 24th Virtual Scientific Sessions!

The 23rd SCMR Scientific Sessions showcased the power of CMR to not only advance our understanding of cardiovascular disease, but to be an important tool in the personalized approach to disease diagnosis and management, one patient at a time. With the upcoming virtual 24th Annual Scientific Sessions from February 18–20, 2021 almost around the corner, we look forward to more transformative science, state of the art lectures, debates, rapid fire cases, and technical innovations (Fig. 6). Check out the SCMR website (www.scmr.org) for futher details!

## Data Availability

Not applicable.

## Abbreviations

AI: Artificial intelligence
BOLD: Blood oxygen level dependent
CIROC: Cardiovascular Imaging Registry of Calgary
CMR: Cardiovascular magnetic resonance
ECV: Extracellular volume fraction
LGE: Late gadolinium enhancement
LV: Left ventricle/left ventricular
LVEF: Left ventricular ejection fraction
MI: Myocardial infarction
RV: Right ventricle/right ventricular
SCMR: Society for Cardiovascular Magnetic Resonance
SPINS: Stress CMR Perfusion imaging in the United States
